# Effect of contrast dose on diagnostic performance in DCE‐MR breast imaging

**DOI:** 10.1002/acm2.13010

**Published:** 2020-10-22

**Authors:** Thuy‐My Thi Le, Elizabeth S. McDonald, Gamaliel Isaac, Mark A. Rosen, Lawrence Dougherty

**Affiliations:** ^1^ Department of Radiology University of Pennsylvania School of Medicine Philadelphia PA USA

**Keywords:** breast, contrast dose, diagnostic performance, magnetic resonance imaging

## Abstract

**Objective:**

To assess the diagnostic performance of breast magnetic resonance (MR) imaging as a function of gadolinium contrast dose using a retrospective reader study.

**Material and Methods:**

IRB approval was obtained prior to the start of this study and was HIPAA compliant. One‐hundred and fifty MR breast examinations were included that were acquired between January 2001 and December 2006. Seventy‐five patients received contrast doses (gadopentetate dimeglumine) by weight of 0.10 mmol/kg and 75 patients were imaged using fixed volumes of 20 ml. The images were assessed by two radiologists with performance calculated for each reader as well as a combined assessment. Dose response was measured by comparing performance between cases binned by dose: <=0.10; >0.10; and >0.13 mmol/kg. Statistical significance was calculated using a one‐sided *Z*‐test for differences in proportions with interobserver agreement calculated using Cohen's kappa statistics.

**Results:**

In the combined reader assessment with equivocal lesions classified as negative, sensitivity rose from 66% (19/29) to 92% (24/26, *P* < 0.01) and 95% (18/19, *P* < 0.01) with the specificity also increasing from 65% (32/49) to 87% (40/46, *P* < 0.01) and 86% (32/37, *P* = 0.01) corresponding to doses <=0.10, >0.10, >0.13 mmol/kg. With equivocal lesions classified as positive, sensitivity rose from 79% (23/29) to 92% (24/26, *P* < 0.10) and 95% (18/19, *P* < 0.10) Specificity also increased from 53% (26/49) to 72% (33/46, *P* < 0.05) and 70% (26/37, *P* = 0.05) with increasing dose. Interobserver agreement also improved at the higher doses.

## 1 INTRODUCTION

Dynamic contrast‐enhanced magnetic resonance imaging (DCE‐MRI) is a common modality of breast cancer detection. However, there have been few studies performed to determine the optimal dose of the gadolinium‐based contrast agents (GBCA). When breast MRI first came into clinical use, many imaging sites used a consistent volume contrast dose of 20 ml (“single dose”). Since maximum conspicuity was desired, the single dose was used regardless of patient weight. Given the range of patient weights typically seen in the United States, this corresponded to a dose range of 0.1 to 0.2 mmol/kg. As the field evolved, there was a desire to standardize the examination across studies, institutions, and manufacturers, particularly for quantification of contrast kinetics. At this point most imaging sites started calculating the dose by weight using 0.1 mmol/kg. Although the American College of Radiology recommends this dose for breast studies^1^ it is not clear how this dose was determined. In the few dose‐response studies that were performed, there is some disparity in conclusions.

Heywang‐Kobrunner et al compared performance using 0.16 mmol/kg gadopentetate dimeglumine versus a low dose of 0.1 mmol/kg of body weight.^2^ The conclusion of this study was that conspicuity of breast lesions was much improved using the higher dose. In a study of the relationship between contrast dose with uptake kinetics using three dose groups (<0.122 mmol/kg; 0.123–0.155 mmol/kg; and> 0.155 mmol/kg), Jansen et al reported that initial and peak enhancement increased with contrast dose for in‐situ and invasive cancers.^3^ However, Knopp et al did not find any improvement in diagnostic accuracy using 0.2 mmol/kg gadopentetate dimeglumine over 0.1 mmol/kg and concluded that a dose of 0.1 mmol/kg was “probably sufficient”.^4^

The reported difference in dose response may be due to different inclusion criteria and diagnosis occurrence rates in each study. For example, in Knopp's study, subjects were recruited who had an “abnormality highly suspicion of being breast cancer” with 75% containing malignancies of which only 5% were DCIS and 8% were ILC.^4^ Jansen's study used only malignant lesions but 33% of the lesions were classified as DCIS.^3^ This suggests that lesions assessed as highly suspicious by mammogram and/or ultrasound will have sufficient conspicuity on MR at low doses of contrast to allow a confident assessment. However, not all cancers respond in the same manner, particularly those with lower angiogenic activity.^5^ In the role that DCE‐MR breast imaging currently fills, (confirmation, staging, and localization), the low dose may be sufficient. However, in screening studies for women at high risk or with dense breasts, in which the highest conspicuity is desired, the optimal dose may be higher than what is currently recommended.

The concern over the safety of gadolinium‐based contrast agents (GBCAs) is an important issue that continues to influence the choice of contrast dose. Nephrogenic systemic fibrosis (NSF) was first described by Grobner et al^6^ and Markmann et al^7^ who reported that patients developed the disease after being exposed to GBCAs. However, upon review of adverse events, the FDA determined that there were no confirmed cases of NSF in patients who had normal kidney function or mild‐to‐moderate kidney insufficiency. Although the report recommended caution when using these agents, GBCAs are safe when patients are adequately screened.^8^ More recently, there has been concern raised over the detection of brain deposits in patients who have received repeated MRI contrast studies^9^, ^10^, ^11^ although the risks, if any, from these deposits is currently unknown. These are important issues and may justify the use of lower GBCA doses. However, before coming to that conclusion, the trade‐off in diagnostic performance should be understood in order to make an informed risk/benefit decision. The purpose of this study was to assess diagnostic performance of breast MR imaging as a function of gadolinium contrast dose using a retrospective reader study of examinations that were acquired during a period of time when the contrast dose was changed from a fixed volume to a weight‐based calculation.

## 2 MATERIALS AND METHODS

### 2.A Patients

IRB approval was obtained prior to the start of this study and was HIPAA compliant. One‐hundred and fifty MR breast examinations were included that were acquired between January 2001 and December 2006, which covered a period before and after the transition from a fixed volume contrast dose to weight‐based calculation of dose. These examinations were chosen because they used similar imaging protocols and had pathologic correlation or 2‐yr follow‐up. Patients were nonpregnant women (mean age 49 yr, range 25–83 yr; mean weight 71 kg, range 49–118 kg) who presented to our institution with palpable or mammographically visible suspicious findings. The MR examinations were not synchronized with the patient's menstrual cycle. Among the 150 examinations, 55 had malignant lesions as determined by biopsy and the remaining 95 examinations were benign or normal. Table 1 shows the distribution of diagnoses for all cases.

**Table 1:** Histologic or 2‐yr follow‐up findings of breast magnetic resonance imaging examinations.

Pathology	All	Dose group ≤ 0.1	Dose group > 0.1	Dose group > 0.13
Benign	95	49	46	37
Adenosis	2	1	1	1
Calcifications	8	5	3	2
Cyst	2	0	2	2
Fibroadenoma	24	11	13	13
Fibrocystic changes	21	9	12	8
Hyperplasia	9	3	6	3
LCIS	4	4	0	0
Papilloma	1	1	0	0
No Lesion	11	8	3	3
Other	13	7	6	5
Cancer	55	29	26	18
DCIS	13	7	6	4
DCIS w/invasive	15	8	7	6
IDC	15	9	6	2
ILC	4	3	1	1
IMC	3	2	1	1
Paget's	1	0	1	1
Phyllodes tumor	2	0	2	1
Other	2	0	2	2
Total	150	78	72	55

Dose in mmol/kg; LCIS, lobular carcinoma in situ; DCIS, ductal carcinoma in situ; IDC, invasive ductal carcinoma; ILC, invasive lobular carcinoma; IMC, invasive mammary carcinoma. Note that the two higher dose groups were formed by setting of the dose threshold so that cases in the >0.13 mmol/kg group were also included in the >0.10 mmol/kg analysis.

### 2.B MR imaging

Images were acquired at 1.5 T using a General Electric Signa (43 examinations) or a Siemens Sonata (107 examinations). Subjects were placed in the prone position with the breasts gently compressed within a dedicated bilateral breast coil. All imaging series were performed in the sagittal plane. T1 and T2 weighted images were acquired followed by a dynamic contrast‐enhanced series. The DCE series was acquired using a radial fast 3D spoiled gradient‐recalled sequence using 512 data samples with 384 projections and 32 slices of 3 mm thickness.^12^ Although this was an IRB‐approved investigational sequence at the time the patient studies were acquired, the protocol can be reproduced with commercial sequences from either manufacturer. Seventy‐five examinations were performed unilaterally, and the remaining 75 cases were bilateral examinations. Scan parameters were as follows: TR = 10 ms; TE = 4 ms; flip angle = 20°–45°. The fat signal was suppressed using a spectral inversion pulse played‐out twice per slice group. A high‐resolution baseline volume was acquired followed by three postcontrast volumes acquisitions over the following 6‐minute period. Contrast (gadopentetate dimeglumine Magnevist, Berlex Laboratories, Wayne, NJ) was administered at 1.5 ml/s followed by a saline flush.

Eighty patients were imaged using a fixed volume gadolinium contrast dose of 20 ml and 70 patients received a dose by weight of 0.1 mmol/kg. The weight of subjects receiving the fixed volume dose ranged from 49 to 118 kg. Five of the patients who received 20 ml of contrast had weights >100 kg yielding doses < 0.1mmol/kg. This created an overall distribution of doses (0.08–0.2 mmol/kg) that spanned changes in the imaging methodology eliminating bias toward a particular scanner or protocol. Among the 43 patients (mean age 49 yr, mean weight 65 kg) performed on the Signa scanner, 35% (15/43) had malignant findings as determined by pathology. Among the 107 patients (mean age 49 yr, mean weight 73 kg) performed on the Sonata scanner, 37% (40/107) had malignant findings.

### 2.C Image assessment

The images were assessed by two radiologists experienced in interpreting breast MR examinations and who were blinded to the biopsy results. One radiologist had 5 yr of experience interpreting breast MR examinations and the second reader was fellowship trained in breast imaging with 12 yr of clinical experience. Assessment was based solely on the MR images consisting of T1 weighted, T2 weighted, and dynamic series morphological appearance and enhancement kinetics. The DCE images were weighted most heavily, with the T2 images useful in cases where the morphology suggested a typically benign lesion such as a fibroadenoma. The T1 images were the least significant and not important except in rare cases such as fat necrosis. The DCE series could be viewed in its original form or with subtraction of the baseline volume. Time vs signal intensity curves from the dynamic series (per pixel, region‐of‐interest (ROI), or whole lesion) were available for interactive viewing. The ROI locations were recorded, which allowed for registration of the detected lesions with pathology reports and also ensured that both readers assessed the same lesion. Clinical assessment was defined as no lesion (BIRADS 1); benign (BIRADS 2); probable cancer (BIRADS 4); definite cancer (BIRADS 5); or indeterminate. To address an indeterminate classification in the clinical setting, the radiologists would have had access to the patient's history, previous imaging results, and/or the option of requesting further testing or surveillance (BIRADS 3). However, for the purposes of this study we required that each examination be classified as benign or malignant. Since the indeterminate classification could be considered either, diagnostic performance was calculated for both and as well with these lesions removed from the analysis. Interobserver agreement was calculated using Cohen's kappa statistics with three diagnostic categories tested for agreement (benign, malignant, and indeterminate lesion classification). During the image read, image quality was scored on a scale of 0–4 corresponding to poor/inadequate, fair/adequate, good, very good, and excellent. Image quality was assessed by considering the signal‐to‐noise ratio, blurring due to patient motion, fat saturation, image shading, streaking, and artifacts. The average score for each dose group was calculated.

Diagnostic performance (sensitivity, specificity, positive predictive value (PPV), negative predictive value (NPV), and overall accuracy) was calculated on a per case basis for each reader as well as a combined assessment for which a true positive by either radiologist scored the case as true positive and a false positive by either reader scored the case a false positive. For examinations with multiple lesions, the correct assessment of any of the lesions as positive scored the case a true positive. For a case in which any positive lesion was incorrectly assessed, then the entire case was scored as false negative, regardless of the of whether the other lesions were correctly identified as true negatives. For bilateral cases, performance was calculated on a per breast basis. Dose response was measured by comparing performance between sets of cases binned by dose: <= 0.10; >0.10; and >0.13 mmol/kg. The >0.13 mmol/kg dose cutoff was chosen after inspection of the initial reader results that showed a predominance of false negatives for cases using doses <=0.13 mmol/kg, with only one false negative at the highest dose. Note that the two higher dose groups were formed by setting the dose threshold; therefore, cases in the highest dose group were also included in the >0.10 mmol/kg analysis. Stratifying the dose groups into more specific bins was not feasible since there would be too few positive cases in each group to achieve significance. Statistical significance was calculated using a one‐sided *Z*‐test for differences in proportions between the low‐dose group and each of the higher dose groups. The one‐sided test was justified since there are no reports that diagnostic performance decreases with increased contrast dose. Statistics were analyzed using Microsoft Excel 2013.

## 3 RESULTS

**Fig. 1:**
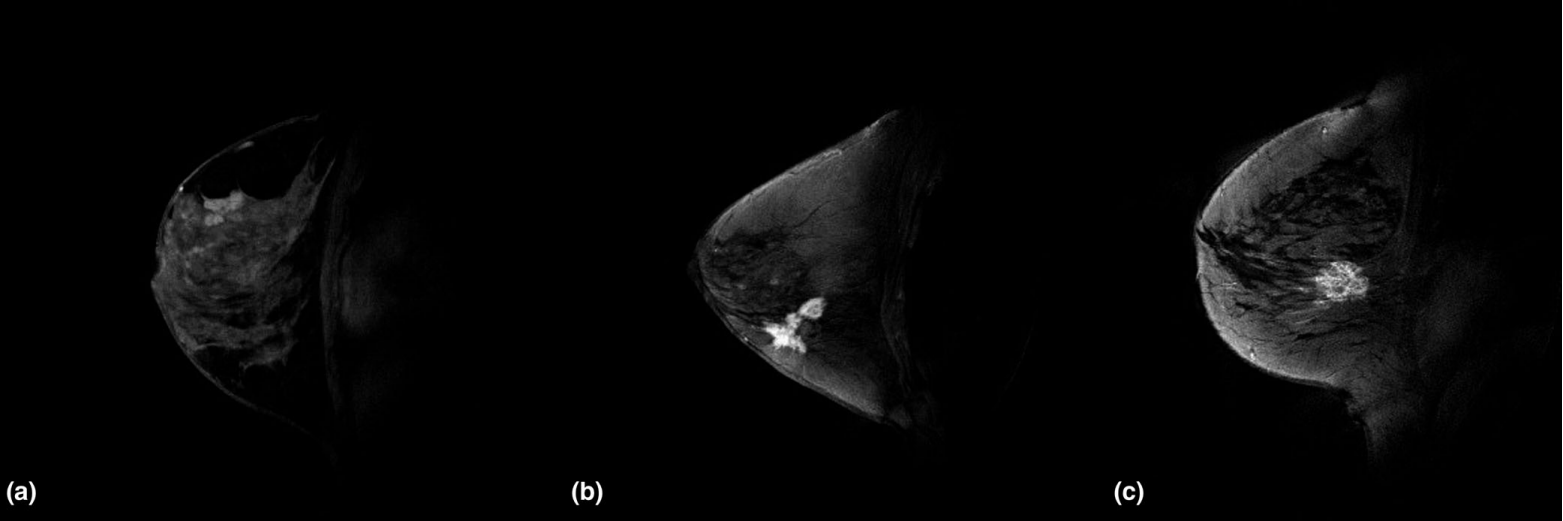
Representative DCE‐MR sagittal images of the breast at different gadolinium contrast doses. (a) 0.10 mmol/kg. (b) 0.13 mmol/kg. (c) 0.15 mmol/kg.

Representative images from positive cases are shown in Fig. 1. Image quality was scored as 2.8 for the low dose and 2.6 for both higher dose groups, which places all three dose groups between good and very good on the quality scale. Diagnostic performance was calculated on a per case basis for both readers as well as a combined assessment. Comparison was made between the low‐dose cases (<=0.10 mmol/kg) and the two higher groups, >0.10 mmol/kg and >0.13 mmol/kg. Interobserver agreement using assessment categories: benign, malignant, or indeterminate at each dose group (low to high) were 0.46, 0.63, and 0.59. Agreement increased when the indeterminate lesions were classified as negative (k = 0.56, 0.79, 0.77). There were 12 false negatives common to both readers of which 10 were in the low‐dose group (DCIS,^5^ ILC,^3^ IDC^2^). The two common false negatives at the higher doses were both DCIS.

Taking lesions classified as indeterminate as a positive finding (Table 2), sensitivity for reader 1 was 72% (21/29) at the low dose and rose to 81% (21/26) and 83% (15/18) at the higher doses, with specificity of 82% (40/49), 78% (36/46), and 78% (29/37). For reader 2, sensitivity increased from 72% (21/29) to 88% (23/26) and 89% (16/18) with specificity also rising from 61% (30/49) to 87% (40/46), and 86% (32/37) at the higher doses. The increased sensitivity for reader 1 was not statistically significant (*P* > 0.2); however, the improved performance for reader 2 showed significance of *P* < 0.10 for the increase in sensitivity and *P* < 0.01 for the increase in specificity. The combined reader assessment showed the sensitivity rising from 79% (23/29) to 92% (24/26, *P* < 0.10) and 94% (17/18, *P* < 0.10) with the specificity also increasing from 53% (26/49) to 72% (33/46, *P* < 0.05) and 70% (26/37, *P* < 0.10).

**Table 2:** Diagnostic performance of DCE‐MR breast imaging as a function of contrast dose — **Equivocal Lesions Positive**

Dose	Reader 1			Reader 2			Combined		
	≤0.1	>0.1	>0.13	≤0.1	>0.1	>0.13	≤0.1	>0.1	>0.13
Sensitivity	72% (21/29)	81% (21/26), *P* = 0.23	83% (15/18), *P* = 0.20	72% (21/29)	88% (23/26), *P* = 0.068	89% (16/18), *P* = 0.089	79% (23/29)	92% (24/26), *P* = 0.086	94% (17/18), *P* = 0.08
Specificity	82% (40/49)	78% (36/46), *P* = 0.67	78% (29/37), *P* = 0.63	61% (30/49)	87% (40/46), *P* = 0.0022	86% (32/37), *P* = 0.012	53% (26/49)	72% (33/46), *P* = 0.030	70% (26/37), *P* = 0.078
PPV	70% (21/30)	68% (21/31), *P* = 0.58	65% (15/23), *P* = 0.64	53% (21/40)	79% (23/29), *P* = 0.011	76% (16/21), *P* = 0.045	50% (23/46)	65% (24/37), *P* = 0.087	61% (17/28), *P* = 0.21
NPV	83% (40/48)	88% (36/41), *P* = 0.28	91% (29/32), *P* = 0.20	79% (30/38)	93% (40/43), *P* = 0.032	94% (32/34), *P* = 0.041	81% (26/32)	94% (33/35), *P* = 0.050	96% (26/27), *P* = 0.041
Accuracy	78% (61/78)	79% (57/72), *P* = 0.44	80% (44/55), p = 0.42	65% (51/78)	88% (63/72), *P* = 0.00077	87% (48/55), *P* = 0.014	63% (49/78)	79% (57/72), *P* = 0.014	78% (43/55), *P* = 0.074

Dose in mmol/kg; PPV, po*P*itive predictive value; NPV, negative predictive value.

**Fig. 2:**
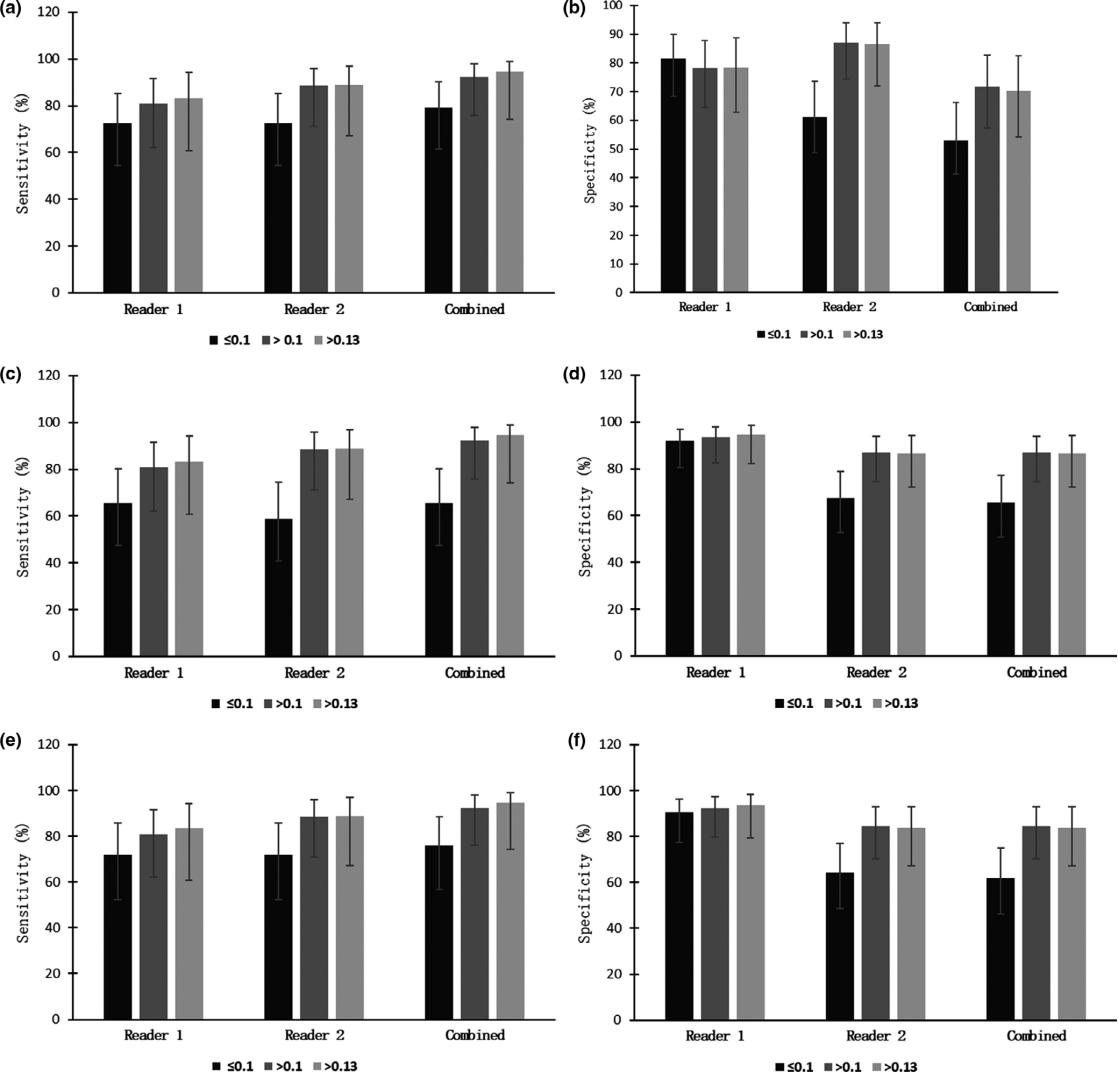
Diagnostic performance as a function of contrast dose. (a,b) Sensitivity and specificity when equivocal lesion were considered a positive finding. (c,d) Sensitivity and specificity when equivocal lesion were considered a negative finding. (e,f) Sensitivity and specificity when equivocal lesion were removed. Performance is shown for each reader and for a combined assessment. Error bars show 95% confidence intervals (Wilson score).

With the indeterminate lesions classified as negative (Table 3), sensitivity for reader 1 was 66% (19/29) for the low dose and increased to 81% (21/26, *P* = 0.10) and 83% (15/18, *P* < 0.10) for the higher doses with specificity of 92% (45/49), 93% (43/46), and 95% (35/37) (no statistical significance for increases). For reader 2, sensitivity increased from 59% (17/29) to 88% (23/26, *P* < 0.01) and 89% (16/18, *P* = 0.01) with specificity also rising from 67% (33/49) to 87% (40/46, *P* = 0.01) and 86% (32/37, *P* < 0.05) at the higher doses. The combined reader assessment showed the sensitivity rising from 66% (19/29) to 92% (24/26, *P* < 0.01) and 94% (17/18, *P* = 0.01) with the specificity also increasing from 65% (32/49) to 87% (40/46, *P* < 0.01) and 86% (32/37, *P* < 0.05). Graphical representations of each reader’s performance are shown in Fig. 2.

**Table 3:** Diagnostic performance of DCE‐MR breast imaging as a function of contrast dose — **Equivocal Lesions Negative**

Dose	Reader 1			Reader 2			Combined		
	≤0.1	>0.1	>0.13	≤0.1	>0.1	>0.13	≤0.1	>0.1	>0.13
Sensitivity	66% (19/29)	81% (21/26), *P* = 0.10	83% (15/18), *P* = 0.09	59% (17/29)	88% (23/26), *P* = 0.0066	89% (16/18), *P* = 0.014	66% (19/29)	92% (24/26), *P* = 0.0081	94% (17/18), *P* = 0.011
Specificity	92% (45/49)	93% (43/46), *P* = 0.38	95% (35/37), *P* = 0.33	67% (33/49)	87% (40/46), *P* = 0.012	86% (32/37), *P* = 0.036	65% (32/49)	87% (40/46), *P* = 0.0069	86% (32/37), *P* = 0.025
PPV	83% (19/23)	88% (21/24), *P* = 0.32	88% (15/17), *P* = 0.30	52% (17/33)	79% (23/29), *P* = 0.011	76% (16/21), *P* = 0.039	53% (19/36)	80% (24/30), *P* = 0.010	77% (17/22), *P* = 0.037
NPV	82% (45/55)	90% (43/48), *P* = 0.13	92% (35/38), *p* = 0.11	73% (33/45)	93% (40/43), *p* = 0.0070	94% (32/34), *P* = 0.016	76% (32/42)	95% (40/42), *P* = 0.0063	97% (32/33), *P* = 0.011
Accuracy	82% (64/78)	89% (64/72), *P* = 0.12	91% (50/55), *P* = 0.14	64% (50/78)	88% (63/72), *P* = 0.00045	87% (48/55), *P* = 0.011	65% (51/78)	89% (64/72), *P* = 0.00034	89% (49/55), *p* = 0.0084

Dose in mmol/kg; PPV, positive predictive value; NPV, negative predictive value

With the indeterminate lesions removed (Table 4), sensitivity for reader 1 was 72% (18/25) for the low dose and increased to 81% (21/26) and 83% (15/18) for high doses with specificity of 90% (38/42), 92% (36/39), 94% (29/31) (no statistical significance for increases). For reader 2, sensitivity increased from 72% (18/25) to 88% (23/26, *P* < 0.10) to 89% (16/18, *P* < 0.10) with specificity increasing from 64% (27/42) to 85% (33/39, *P* = 0.01) and to 84% (26/31, *P* = 0.05) at high doses. The combined reader assessment showed sensitivity rising from 76% (19/25) to 92% (24/26, *P* = 0.05) and 94% (17/18, *P* = 0.05) with specificity increasing from 62% (26/42) to 85% (33/39, *P* = 0.1) to 84% (26/31, *P* < 0.05).

**Table 4:** Diagnostic performance of DCE‐MR breast imaging as a function of contrast dose — **Equivocal Lesions Removed**

Dose	Reader 1			Reader 2			Combined			
	≤0.1	>0.1	>0.13	≤0.1	>0.1	>0.13	≤0.1	>0.1	>0.13
Sensitivity	72% (18/25)	81% (21/26), *P* = 0.23	83% (15/18), *P* = 0.19	72% (18/25)	88% (23/26), *P* = 0.069	89% (16/18), *P* = 0.090	76% (19/25)	92% (24/26), *P* = 0.055	94% (17/18), *P* = 0.053
Specificity	90% (38/42)	92% (36/39), *P* = 0.38	94% (29/31), *P* = 0.34	64% (27/42)	85% (33/39), *P* = 0.018	84% (26/31), *P* = 0.051	62% (26/42)	85% (33/39), *P* = 0.011	84% (26/31), *P* = 0.036
PPV	82% (18/22)	88% (21/24), *P* = 0.29	88% (15/17), *P* = 0.29	55% (18/33)	79% (23/29), *P* = 0.019	76% (16/21), *P* = 0.065	54% (19/35)	80% (24/30), *P* = 0.014	77% (17/22), *P* = 0.051
NPV	84% (38/45)	88% (36/41), *P* = 0.32	91% (29/32), *P* = 0.25	79% (27/34)	92% (33/36), *P* = 0.072	93% (26/28), *P* = 0.083	81% (26/32)	94% (33/35), *P* = 0.050	96% (26/27), *P* = 0.047
Accuracy	84% (56/67)	88% (57/65), *P* = 0.25	90% (44/49), *P* = 0.23	67% (45/67)	86% (56/65), *P* = 0.0050	86% (42/49), *P* = 0.041	67% (45/67)	88% (57/65), *P* = 0.0024	88% (43/49), *P* = 0.025

Dose in mmol/kg; PPV, positive predictive value; NPV, negative predictive value.

## 4 DISCUSSION

These data show there is an increase in sensitivity for malignancy with increasing contrast dose. There was no significant decrease in specificity and a substantial improvement in the second reader's discrimination of benign lesions with the higher doses. Greater confidence in the lesion assessment was also shown by the interobserver agreement, which increased at the higher doses. Most of the false negatives common to both readers were in the low‐dose group and represented either DCIS or ILC. This supports that higher contrast doses can increase the contrast‐to‐noise ratio (CNR) of lesions with lower or inconsistent enhancement, aiding sensitivity and improving specificity as well. The image quality score was consistent across dose groups showing that there was no bias in the results from technical issues or subject demographics.

The first reader classified more lesions as indeterminate than did the second reader and how those were interpreted had a large effect on performance measures. In the true clinical setting, equivocal lesions would not be simply classified as a group to be benign or malignant but could instead be followed to assess changes. Additional information from other examinations could also make a definitive diagnosis possible in many cases. Therefore, many of these would subsequently be correctly classified as benign or malignant and the performance measures would be higher. However, regardless of whether the indeterminate lesions were classified as positive, negative to removed altogether, reader performance improved at the higher contrast doses.

Our study had limitations. Although these results indicate that a dose higher than 0.1 mmol/kg would give better diagnostic performance, the specific optimal dose was not determined since there were no enough cases at each dose level to show significance. More studies, particularly positive cases, performed at various contrast doses would allow stratification of the doses giving a better indication of which was optimal. More significant results could have been achieved if the studies had been repeated on the same patient using different doses. Since this was a retrospective study, that was not an option. Another limitation was that only one contrast agent (gadopentetate dimeglumine) was used in the study. Investigation of the dose response using newer compounds with higher relaxivity would be helpful since they can improve CNR at lower volumes. However, these are often used in the reverse manner, lowering the concentration while maintaining the CNR that was achieved with the older compounds at 0.1 mmol/kg. This study suggests that there would be a benefit to taking advantage of the higher CNR that can be achieved using these agents.

The safety of GBCAs may be the overriding factor in choosing the dose, but the trade‐off in diagnostic performance should be understood in order to make an informed decision. This study showed that the diagnostic performance of DCE‐MR breast imaging was greater when subjects received a higher gadolinium dose than the current standard of 0.10 mmol/kg.

## CONFLICT OF INTEREST

None of the authors has any conflict of interest. There is no industry support for this project.
